# Identification of Aggregation Processes in Hexamethylenetetramine Aqueous Solutions: A Comprehensive Raman and Acoustic Spectroscopic Study Combined with Density Functional Theory Calculations

**DOI:** 10.3390/molecules28237838

**Published:** 2023-11-29

**Authors:** Stefanos Tsigoias, Michael G. Papanikolaou, Themistoklis A. Kabanos, Angelos G. Kalampounias

**Affiliations:** 1 Physical Chemistry Laboratory, Department of Chemistry, University of Ioannina, 45110 Ioannina, Greece; 2 Section of Inorganic and analytical Chemistry, Department of Chemistry, University of Ioannina, 45110 Ioannina, Greece; 3 Institute of Materials Science and Computing, University Research Center of Ioannina (URCI), 45110 Ioannina, Greece

**Keywords:** Raman, ultrasonic relaxation spectroscopy, molecular orbital calculations, hexamethylenetetramine, aggregation mechanism

## Abstract

Raman scattering has been employed to study in detail the concentration dependence of the vibrational modes for hexamethylenetetramine (HMTA) aqueous solutions. The formation of protonated and/or aggregated species has been clarified by comparing the experimental with the theoretically predicted vibrational spectra by means of quantum mechanical calculations. The analysis has shown that the vibrational modes of the solutions arise from a contribution of the vibrational modes of the HMTA self-aggregates and hetero-aggregates of HMTA with water molecules that are formed in the low- and intermediate-concentration regions, respectively. The protonation of HMTA is ruled out due to the large differences between the experimental and the theoretically calculated spectra of the protonated molecules of HTMA in the fingerprint region. In the low-concentration solutions, the hetero-aggregation reaction of HMTA with water is the dominant mechanism, while at higher concentrations, a self-aggregation mechanism occurs. Ultrasonic absorption and velocity measurements were carried out for hexamethylenetetramine aqueous solutions. The acoustic spectra reveal the presence of only one single Debye-type relaxation process that is assigned to the aggregation mechanism of HMTA. The sound absorption data follow two different dependencies on the HMTA mole fraction. The crossover 0.018 mole fraction signifies two separate regions with distinct structural characteristics. The relaxation mechanism observed in dilute solutions was attributed to hetero-association of HMTA with water molecules, while at higher concentrations, the observed relaxation process was assigned to the self-association reaction of HMTA molecules. This structural transformation is also reflected in several physicochemical properties of the system, including the kinematic viscosity, the mass density, the sound speed and the adiabatic compressibility of the HMTA aqueous solutions. The combination of vibrational and acoustic spectroscopies with molecular orbital calculations allowed us to disentangle the underlying processes and to elucidate the observed relaxation mechanism in the HMTA aqueous solutions.

## 1. Introduction

Hexamethylenetetramine (HMTA) is a tertiary polyamine, whose structure resembles that of a cage. HMTA is also known as methenamine, hexamine or urotropine. Hexamethylenetetramine is used in medicine for the treatment of urinary tract infections (UTIs) and it is effective as a daily low-dose antibiotics at preventing UTIs [1]. It is also used for heating camping food or military rations, since it burns without smoke and it has a high energy density, it does not liquify while burning and leaves no ashes (although its fumes are toxic) [2]. The wide range of applications also includes its use as a food additive/preservative corresponding to the International Numbering System for Food Additives (INS number) 239 [3]. HMTA is used as the base component to produce a variety of highly explosive organic compound including RDX (abbreviation of Research Department eXplosive), C-4, octogen, HMTD (Hexamethylene Triperoxide Diamine) and others [4]. Finally, HMTA is a versatile reagent in organic synthesis [5], while its dominant use is in the production of powdery or liquid preparations of phenolic resins, where it is added as a hardening component [4].

Hexamethylenetetramine is a heterocyclic organic compound with a tetrahedral cage-like structure similar to adamantane. Four vertices are occupied by nitrogen atoms that are connected by methylene groups. Despite the molecular cage-like shape of HTMA, at the interior of the molecule no void space is available to incorporate other atoms or smaller molecules. The HMTA molecule behaves like an amine base, undergoing protonation and molecular self- and hetero-aggregation reactions with solvent molecules. Its rigid molecular structure does not permit any conformational change.

A deep understanding of HMTA’s structural and dynamical properties is essential for predicting several macroscopic properties of this amine that may have important implications in its already wide range of applications. The combination of vibrational (Raman) spectroscopy with acoustic relaxational spectroscopy has been proven to be a powerful tool in investigating the association–dissociation [6,7] and the proton-transfer [8,9] mechanisms, and generally in reaction engineering [10]. Ultrasonic relaxation spectroscopy may provide dynamical and structural information about the studied system in the frequency domain covering a wide range from 10 kHz to ∼10 GHz that corresponds to relaxation times between 10^−5^ and 10^−11^ s, without any restriction on the type of process studied [11,12,13,14]. Additionally, Raman spectroscopy provides vibrational relaxation and structural information at the molecular level in a time scale of 10^−12^ to 10^−14^ s. By combining Raman with ultrasonic relaxation spectroscopy, one can provide a complete and frank account for the physicochemical behavior of the system. On the other hand, Density Functional Theory (DFT) calculations can be used to help us disentangle the underlying processes and to elucidate the observed relaxation mechanism in the acoustic spectra.

Our aim in this study was to provide insights on the structure and dynamics of aqueous solutions of HMTA by performing vibrational and acoustic concentration-dependent measurements. The possibility of formation of protonated and/or aggregated species was clarified by comparing the experimental spectroscopic data with quantum mechanical calculations on isolated species in the gas phase free of any interactions.

## 2. Results and Discussion

### 2.1. Structural Processes in HMTA Aqueous Solutions

The HMTA molecule acts as an amine base, and thus, it may undergo protonation reactions with solvent molecules. The proton-transfer mechanism can be described as:

(1) $$HMTA+zH_{2}O⇄{HMTA}^{+z}+z{OH}^{-}$$

**Figure 1:**
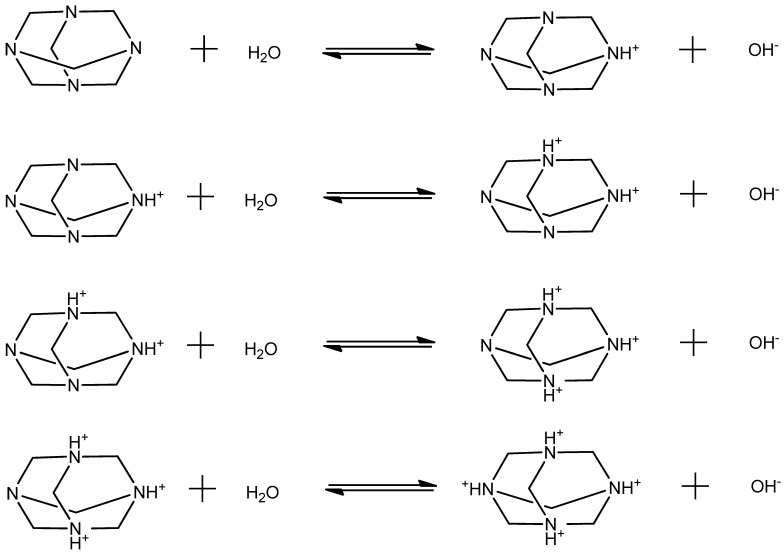
Possible proton-transfer reactions that may occur in HMTA aqueous solutions. Each reaction corresponds to a subsequent protonation step. The protonation constant of HMTA is pKa = 4.89. HMTA is a weak base and only one of its nitrogen atoms tends to be protonated, while the rest are expected to be hydrogen bonded with water molecules as shown in [5].

The possible proton-transfer reactions that may occur in HMTA aqueous solutions are shown in Figure 1. Each reaction corresponds to a subsequent protonation step.

The molecular structure of HMTA permits not only a molecular hetero-aggregation, but also a self-aggregation reaction. In addition, other processes such as a proton transfer reaction of HMTA with the solvent (water) may also occur. Processes related to conformational changes are unlikely to occur due to the rigid molecular structure of HMTA.

The hetero-aggregation reaction of HMTA with water is likely to occur at low concentrations of amine, where the solute–solvent interactions are expected to be stronger, and can be described through the following scheme:

(2) $${\left(H_{2}O\right)}_{m}+HMTA⇄HMTA\cdot mH_{2}O$$

**Figure 2:**
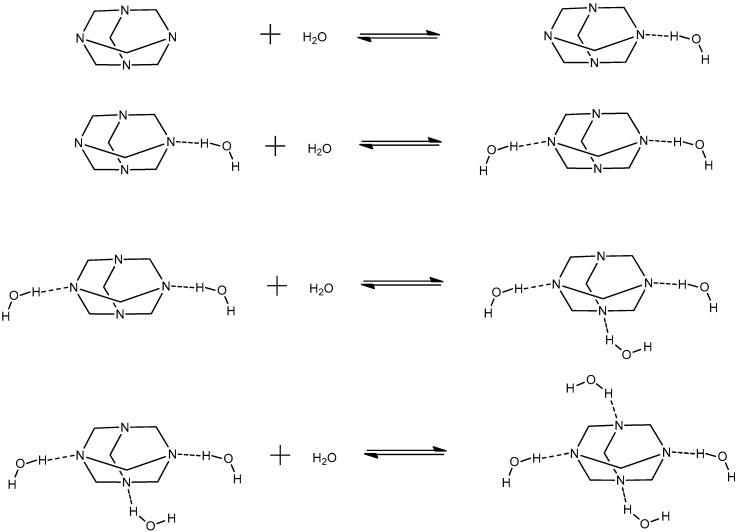
Possible hetero-aggregation reactions that may occur in HMTA aqueous solutions. Each reaction corresponds to a subsequent aggregation step.

The (H_2_O)_m_ and HMTA ∙ mH_2_O complexes denote the corresponding water clathrate and the mixed aggregate between water and HMTA molecules, respectively, and both are constituted through hydrogen bonds [15,16,17,18]. Parameter m is the number of water molecules that are associated in the formation of the water clathrate and mixed aggregate. The possible hetero-aggregation reactions that may occur in HMTA aqueous solutions are presented schematically in Figure 2. Each reaction corresponds to a subsequent aggregation step.

**Figure 3:**
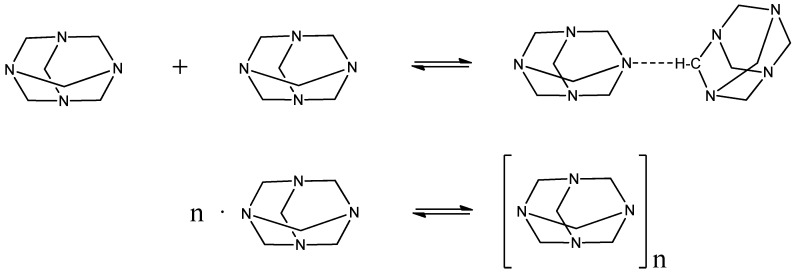
Possible self-aggregation reactions that may occur in HMTA aqueous solutions. The second reaction corresponds to a mean reaction of the usually stepwise aggregation mechanism. This hypothesis is valid for relatively low values of the aggregation number n.

At intermediate concentrations of HMTA aqueous solutions, a self-aggregation reaction of HMTA may occur, which can be described as:

(3) $$nHMTA⇄{\left(HMTA\right)}_{n}$$

with n designating the aggregation number and (HMTA)_n_ the HMTA aggregate. The self-aggregation mechanism is usually a stepwise reaction [19]. Nevertheless, for low values of the aggregation number, this reaction can be approximated as a mean mechanism that is described by Equation (3). The possible self-aggregation reactions that may occur in HMTA aqueous solutions are illustrated in Figure 3.

**Figure 4:**
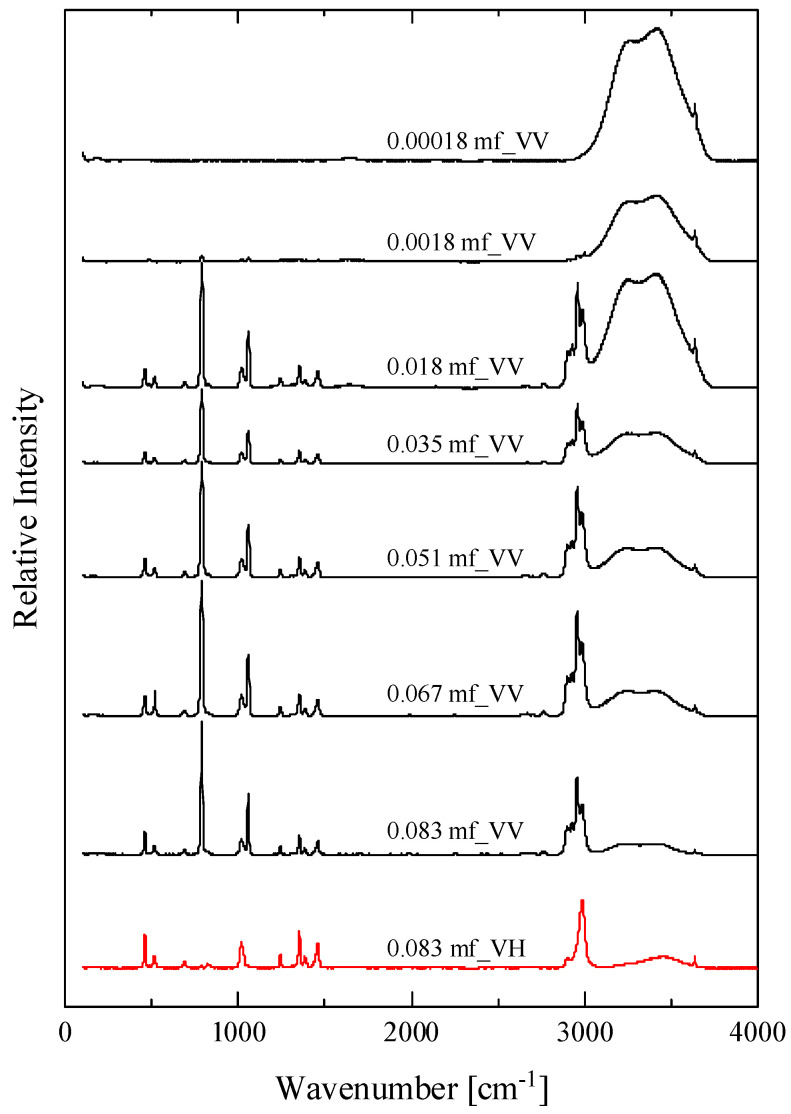
Concentration dependence of the experimental polarized (VV) Raman spectra of HMTA aqueous solutions in the dense concentration region at 20 °C. The depolarized (VH) spectrum is presented only for one solution corresponding to a 0.083 mole fraction of HMTA. The intensity of the depolarized spectrum is multiplied by a factor of 2 to facilitate the observation of the bands in the fingerprint region (<1500 cm^−1^).

In an effort to verify which mechanism occurs upon dilution of HMTA in water, we recorded the Raman spectra of solutions in the dense concentration region at 20 °C. The polarized (VV) spectra in the 0.00018 to 0.083 mole fraction region are presented in Figure 4. The depolarized (VH) spectrum is shown only for the solution with a 0.083 mole fraction of HMTA. No significant spectral changes were observed with varying the HMTA mole fraction in the solutions. At mole fractions below 0.0018, the spectra were dominated by the presence of the solvent bands, especially in the frequency range above 3000 cm^−1^. The bands observed in the fingerprint region, that is below 1500 cm^−1^, are due to CNC deformation and stretching, CN stretching and CH_2_ deformation, twisting and wagging vibrations [20,21]. In the 2800–3000 cm^−1^ region, the high-intensity bands were assigned to the symmetric and asymmetric C-H stretching modes, while above 3000 cm^−1^, the spectrum was dominated by the presence of broad and strongly overlapping bands that were attributed to the O-H stretching modes of the hydroxyl functional groups that belong to water molecules. We also detected a relatively sharp band near ~3636 cm^−1^, which was assigned to the O-H stretching modes of the hydroxyl of water molecules that are hydrogen bonded with HMTA molecules. As the solutions became more concentrated with the amine, the intensity of the ~3636 cm^−1^ peak decreased, but it continued to exist. This means that even in the concentrated amine solutions, HMTA complexation with water takes place. From the depolarized spectrum, it seems that the 785 cm^−1^ band, assigned to the stretching mode of the CNC with A_1_ symmetry, was strongly polarized. The rest of the bands were only partially polarized. The experimental and theoretically estimated Raman frequencies of HMTA in solution and in vacuum, respectively, as well as a tentative assignment are presented in Table 1. The experimental frequency of the CNC deformation vibration differs by 4 cm^−1^ with the corresponding frequency of the calculated spectra, which is reasonable since the calculation was performed in the vapor state and not in solution state where intermolecular interactions are present.

Depending on the concentration of HMTA in aqueous solutions, HMTA can strengthen or destroy the hydrogen bonding network of water. In dilute solutions, HMTA seems to strengthen the hydrogen bonding network of water by binding with it. In the higher concentration region of HMTA, the rigidity of the clathrate network of water through hydrogen bonding tends to collapse, since HMTA starts to aggregate with itself as well as with water.

**Figure 5:**
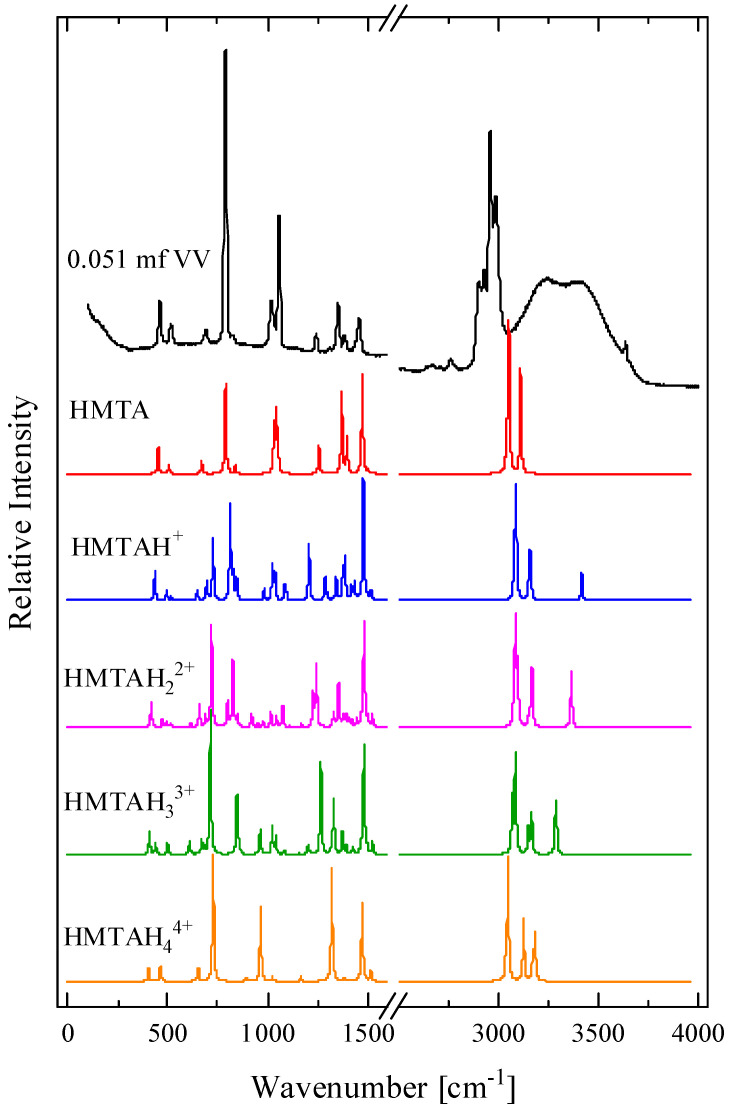
The calculated spectra of HMTA protonated species with one, two, three and four protons. See text for details concerning the calculation procedure. The experimental polarized Raman spectrum of the HMTA solution corresponding to a 0.051 mole fraction is also presented for comparison.

In Figure 5, the calculated spectra of HMTA protonated species with one, two, three and four protons are illustrated. The experimental polarized (VV) Raman spectrum of the HMTA solution corresponding to a 0.051 mole fraction is also presented for direct comparison. The results indicate that we can rule out the protonation of HMTA due to the large differences between the experimental and the theoretically calculated spectra of the protonated molecules of HTMA. The spectra of the protonated molecules of HMTA yielded many more peaks compared to the experimental spectrum, especially in the fingerprint region.

**Figure 6:**
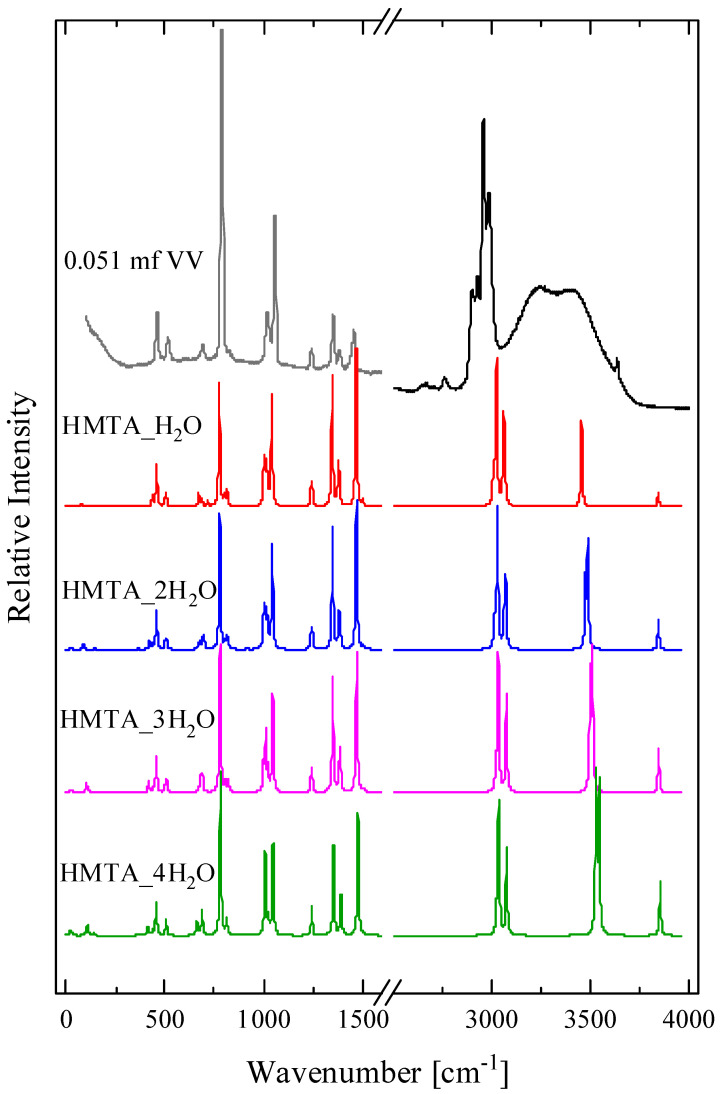
The calculated spectra of HMTA aggregated species with one, two, three and four water molecules. See text for details concerning the calculation procedure. The experimental polarized Raman spectrum of an HMTA solution corresponding to a 0.051 mole fraction is also presented for comparison.

In Figure 6, we present the calculated spectra of HMTA aggregated species with one, two, three and four water molecules. The experimental polarized (VV) Raman spectrum of an HMTA solution corresponding to a 0.051 mole fraction is again shown for comparison reasons. Almost all bands coincided in frequency, while they slightly differed only in their relative intensities in the low-frequency region. The results reveal that the theoretical findings are in close agreement with the experimental Raman spectrum corresponding to a 0.051 mole fraction even though the calculation was carried out in the vapor state. In addition to the very good identification of the experimental spectrum with the theoretically calculated ones in the range from 250 to 1500 cm^−1^, we also identified a peak at ~3636 cm^−1^ of the experimental spectrum, which was attributed to the O-H stretching mode of the water molecule that is hydrogen bonded to the HMTA amine molecule. The reason why this peak appeared at higher wavenumbers in the theoretical compared to the experimental spectrum is because all the theoretical spectra were calculated in the gas phase. This is also the reason why the C-H vibrations were also shifted to higher wavenumbers in the theoretical spectra. The CH_2_ groups are in the outer space of the molecule, and as such, are the most strongly affected by the presence of other neighboring molecules. The peak observed slightly above 3500 cm^−1^ in the theoretical spectra was attributed to the N-H stretching vibration of the hydrogen bond with water. In the experimental spectra, this peak was not detected probably because it is overwhelmed by the spectrum of water in that frequency region. The main differences between the experimental Raman spectra and the calculated spectra of the protonated species can be seen in the 250–1500 cm^−1^ spectral range. The theoretically predicted spectra of the protonated species exhibited many more vibrational modes in the specific spectral range compared to the experimental spectrum, which leads to the conclusion that the protonated species are not present in the solutions. Even the spectrum of the monoprotonated molecule of HTMA did not reveal a close resemblance with the experimental spectra and this is expected considering the low basicity of HMTA amine. Furthermore, the extra peaks observed in the spectra of the protonated species were not present in the calculated spectrum of HMTA·20H_2_O.

**Figure 7:**
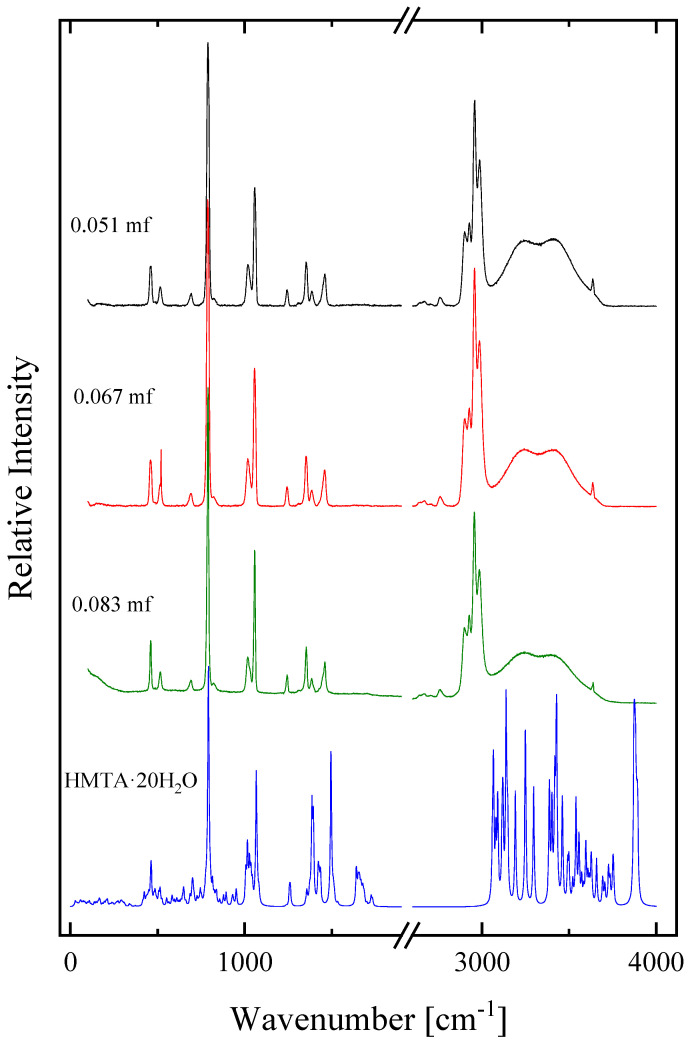
The calculated spectrum of HMTA aggregated species with twenty water molecules. See text for details concerning the calculation procedure. The HMTA·20H_2_O aggregate closely mimics the experimental solution with a 0.051 mole fraction (molality equal to 3), in which the HMTA:H_2_O molar ratio is 1:18.5. The experimental polarized Raman spectra of three different HMTA solutions corresponding to 0.051, 0.067 and 0.083 mole fractions are also presented for comparison.

In Figure 7, the calculated spectrum of the HMTA·20H_2_O aggregate species with twenty water molecules is presented. This aggregate was chosen to be studied theoretically because it closely mimics the experimental solution with a 0.051 mole fraction (molality equal 3), in which the HMTA:H_2_O molar ratio is 1:18.5. The experimental polarized Raman spectra of three HMTA solutions corresponding to 0.051, 0.067 and 0.083 mole fractions are also presented for comparison.

Also in this case, a very good resemblance was observed between the experimental and theoretical spectrum in the fingerprint region. Furthermore, the intense peak observed in the highest frequency of the HMTA·20H_2_O aggregate’s theoretical spectrum was attributed to the O-H stretching mode of the water molecule that is hydrogen bonded to the HMTA amine molecule and is related with the peak located at ~3636 cm^−1^ in the experimental spectrum. This peak appeared at higher wavenumbers because the calculation was performed in the gas phase without considering any intermolecular interactions. The same also holds for the theoretically predicted frequencies of the C-H vibrations that were also calculated at higher wavenumbers relative to the experimental frequencies. Based on this more complex theoretical calculation, we can safely attribute this peak to the hydroxyl groups of the water molecules that are in the outer part of the aggregate. One hydrogen atom of these water molecules is bound to another water molecule of the aggregate, while the second hydrogen atom is free and not bounded to other water molecules of the aggregate.

**Figure 8:**
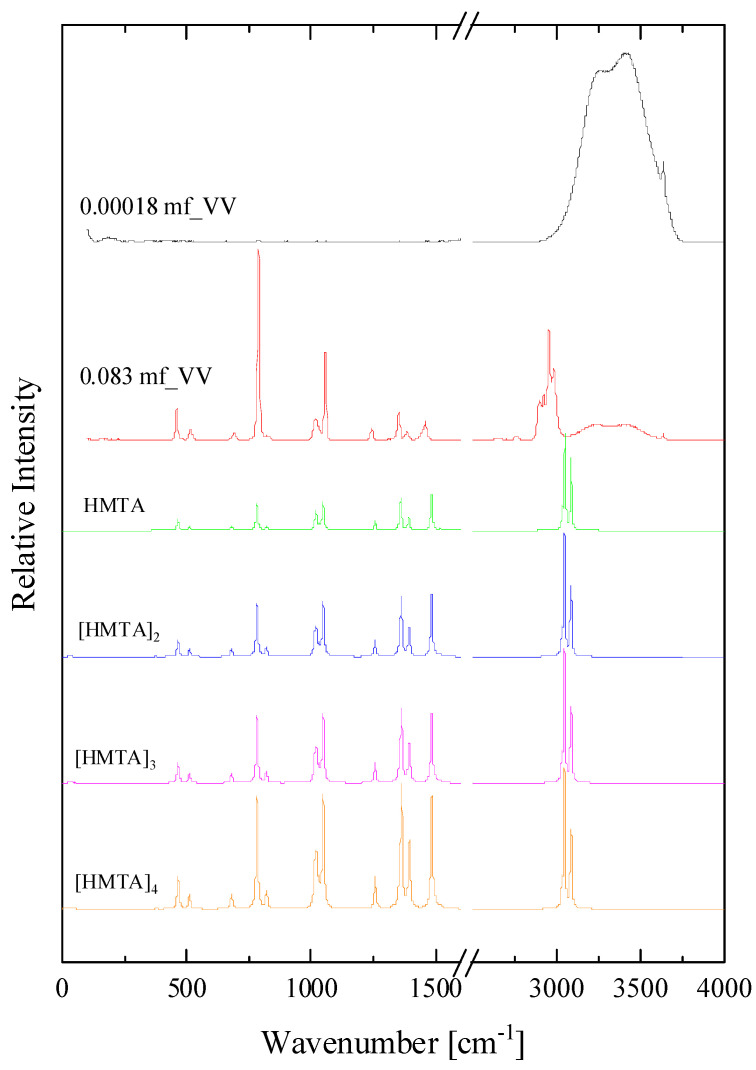
The calculated spectrum of HMTA self-aggregated species with aggregation number n equal to one, two, three and four. See text for details concerning the calculation procedure. The experimental polarized Raman spectra of two HMTA solution with 0.00018 and 0.083 mole fractions corresponding to low and intermediate concentrations are also presented for comparison.

Let us now examine if a self-aggregation of HMTA is likely to occur at higher mole fractions. We present in Figure 8, the calculated spectrum of HMTA self-aggregated species with aggregation number n equal to one, two, three and four. The experimental polarized (VV) Raman spectra of two HMTA solutions with 0.00018 and 0.083 mole fractions corresponding to two limiting concentrations are also presented for comparison. We observe an adequate matching between the experimental spectrum of the concentrated amine solution with the theoretically calculated spectra for the self-aggregation reaction between HMTA molecules (see Table 1). These results indicate that a self-aggregation mechanism may occur upon dilution of HMTA in an aqueous environment.

From the above discussion, we can conclude that in the dilute solutions, the dominating mechanism is the hetero-aggregation reaction of HMTA with water, while at higher mole fractions, a self-aggregation mechanism occurs. Experimental evidence for the protonation of HMTA was not found.

### 2.2. Sensing of the Aggregation Processes via Ultrasonic Absorption Measurements

The energy of an acoustic wave travelling in a medium is absorbed and is transformed to heat when the density and sound pressure fluctuations are out of phase. In the case of homogeneous liquids, the mechanisms behind this process are known as relaxation mechanisms and originate from the time lag observed in the perturbation of a physical or chemical equilibrium started by a cyclical wave parameter fluctuation. The total energy of the acoustic wave is distributed among the translational, the vibrational and the structural states and its redistribution is strongly related to the coupling processes taking place in the medium that ultrasound propagates. These coupling processes set up the number and the type of absorption mechanisms observed in every liquid. Ultrasonic relaxation spectroscopy is one of the most powerful techniques in investigating the aggregation phenomena taking place not only in neat liquids but also in solutions, providing information concerning kinetics and thermodynamic properties. In aqueous solutions, the volume change taking place due to aggregation reactions is the key factor for the coupling between an acoustic wave and a chemical equilibrium. Aggregation phenomena may occur on a variety of length scales from tenths of Å to nm and time scales from ns to s.

The sound absorption coefficient *a* for a non-electrolytic system is given by [22,23]:

(4) $${\left(\frac{a}{f^{2}}\right)}_{total}={\left(\frac{a}{f^{2}}\right)}_{relaxing\;part}+{\left(\frac{a}{f^{2}}\right)}_{non-relaxing}$$

The first term of the sum is related to the relaxing part that is frequency-dependent and is associated with the relaxation processes that may be present in the system studied. The second term is related with the non-relaxing or classical part, which is frequency-independent, at least in the frequency range where the measurements are performed. The so-called non-relaxing term is also labeled with B and includes contributions from several co-existing effects such as the vibrational relaxation, the viscous absorption, the thermal absorption and the radiation:

(5) $$B={{\left(\frac{a}{f^{2}}\right)}_{vibrational}+{\left(\frac{a}{f^{2}}\right)}_{viscous}+\left(\frac{a}{f^{2}}\right)}_{thermal}+{\left(\frac{a}{f^{2}}\right)}_{radiation}$$

Usually, the contribution of the latter is ignored because it is insignificant relative to the other three contributions. The sound absorption coefficient due to vibrational relaxation is a linear function of temperature and appears constant when the system is at a given temperature under thermodynamic equilibrium. Viscous contribution to the sound absorption or Stokes term is given by:

(6) $${\left(\frac{a}{f^{2}}\right)}_{viscous}=\left(\frac{2\pi^{2}}{3\rho u^{3}}\right)\left(3\eta_{V}+4\eta_{S}\right)$$

with *η_V_* and *η_S_* denoting the shear and volume viscosities, respectively. Other symbols have their usual meanings, that is density *ρ* and sound velocity *u*. The third contribution is the thermal sound absorption or Kirchhoff term and is a function of several thermodynamic parameters including thermal conductivity *Q* of the solution and the specific heats under constant pressure *C_p_* and volume *C_p_/C_v_*. The equation for the estimation of the thermal losses is:

(7) $${\left(\frac{a}{f^{2}}\right)}_{thermal}=\left(\frac{2\pi^{2}}{\rho u^{3}}\right)\frac{\left(\gamma -1\right)Q}{C_{P}}$$

where *γ* is the ratio *C_p_/C_v_*. For most systems, thermal losses may be considered as negligible relative to the viscous losses and in general *B* is frequency-independent [22,23].

For simple liquids, the sound absorption coefficient can be fitted in the frequency domain by a single Debye-type equation through the equation [22,23,24,25]:

(8) $$\frac{a}{f^{2}}=\frac{A}{1+{\left(\frac{f}{f_{r}}\right)}^{2}}+B$$

with *f_r_* denoting the characteristic relaxation frequency of the process. The corresponding relaxation time of the process is given by:

(9) $$\tau_{r}=\frac{1}{2\pi f_{r}}$$

In the *a/f^2^* vs. *f* semi-log plot, the classical absorption *B* is observed as a straight line, while the relaxation process appears as an excess sigmoidal form over this straight line. The single Debye-type process appears as a simple exponential relaxation in the time-domain.

**Figure 9:**
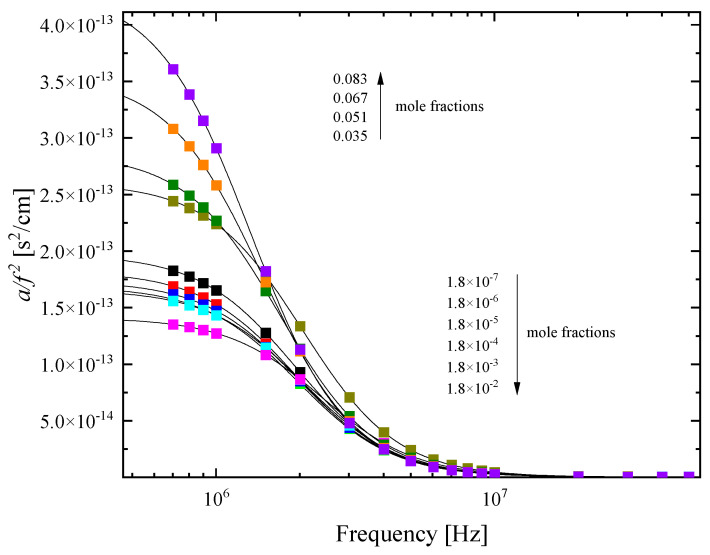
Ultrasound absorption spectra in the frequency reduced form (*a/f^2^*) for all concentrations of HMTA aqueous solutions at 20 °C. Squared symbols represent experimental points and solid lines denote the single Debye-type relaxation profiles for each concentration. See text for more details concerning the fitting of the experimental spectra. The ultrasound absorption coefficient of the solvent (water) was constant in the MHz frequency range studied in this study. Arrows denote that the relaxation amplitude increases in the range of 0.035–0.083 and decreases in the range of 1.8 × 10^−7^–1.8 × 10^−2^.

The absorption spectra in the frequency reduced form (*a/f^2^*) as a function of frequency for all concentrations of HMTA aqueous solutions at 20 °C are shown in Figure 9. The symbols represent experimental points, while the solid sigmoidal lines denote the single Debye-type relaxation profiles for each concentration. The solvent ultrasound absorption coefficient was found to be constant in the MHz frequency range studied in this study and equal to ~20 × 10^−17^ s^2^/cm. The results reveal that the relaxation amplitude increased in the mole fraction range of 0.035–0.083 and decreased in the range of 1.8 × 10^−7^–1.8 × 10^−2^. The trend of the relaxation amplitude is shown by arrows in the graph. The fitting using Debye-type profiles was adequate to fit all the experimental spectra as indicated by the goodness of fit observed in Figure 9. The single relaxation mechanism observed in the acoustic spectra of the HMTA aqueous solutions was attributed to the aggregation reaction, which is clearly a structural process. These processes are related to shear (structural) viscosity and dominate in strongly associated solutions and neat liquids consisting of polar molecules, such as alcohols, amines, water, etc., that are bonded with hydrogen bonds.

**Figure 10:**
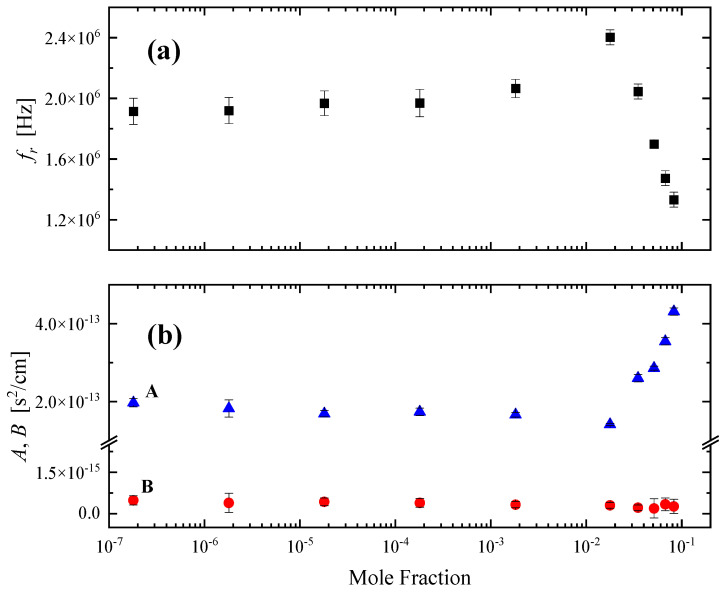
(**a**) Characteristic ultrasonic relaxation frequency *f_r_* as a function of the mole fraction for HMTA aqueous solutions at 20 °C. (**b**) Concentration dependence of the relaxation amplitude *A* and classical contribution to *a/f^2^* denoted as *B*.

The characteristic relaxation frequency *f_r_*, the amplitude of the relaxation *A* and the classical ultrasound absorption *B* were the only free fitting parameters in the non-linear least-mean square fitting procedure and their values as a function of the HMTA mole fraction are presented in Figure 10a,b.

As already discussed, the association mechanism is two-fold. Amine molecules form structures with several water molecules in the region of low concentrations and amine molecules form structures of different sizes ranging from monomers to clusters of monomers at intermediate concentrations. As is evident from the experimental acoustic spectra, all steps of the association reaction are detected as one “mean” Debye-type relaxation. It is interesting to note that the relaxation frequency experienced a drastic change below and above a 0.018 mole fraction of HMTA. Initially, the characteristic frequency slightly increased, while above this mole fraction suddenly decreased. An analogous behavior was demonstrated by the relaxation amplitude in the same mole fraction. The only difference between the two acoustic parameters is that they exhibited exactly opposite trends. Furthermore, for each acoustic parameter, the rates below and above the 0.018 mole fraction were quite different.

The crossover 0.018 mole fraction signifies two separate regions with distinct structural characteristics. The relaxation observed in dilute solutions was attributed to the hetero-association of HMTA with water molecules, while at higher concentrations, the observed relaxation was assigned to the self-association reaction of HMTA molecules. The behavior demonstrated in Figure 10 was assigned as a “spectral signature” of the system’s structural transition from hetero- to self-aggregation of HMTA. Indeed, the amplitude of the relaxation initially decreased with concentration and then suddenly increased, implying the enhancement of the self-aggregation of HMTA at the expense of the hetero-aggregation mechanism of HMTA with water molecules which was stronger in the low concentration limit. The classical contribution *B* to the ultrasound absorption coefficient per squared frequency *a/f^2^* appeared to be almost constant for all solutions, as expected.

**Figure 11:**
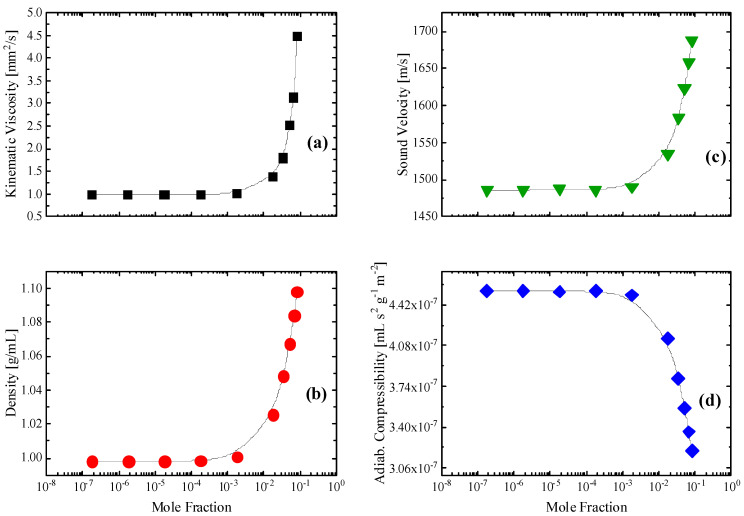
Concentration dependence of the kinematic viscosity (**a**), mass density (**b**), ultrasound velocity (**c**) and adiabatic compressibility (**d**) for all HMTA aqueous solutions studied. All physical properties and acoustic parameters were measured at 20 °C. Lines denote the two distinct regions of interest.

The structural model proposed for the HMTA aqueous solutions considering intermolecular interactions is expected to be reflected in several physicochemical properties of the system. In Figure 11a–d, we present the concentration dependence of the kinematic viscosity, the mass density, the sound speed and the adiabatic compressibility of all HMTA aqueous solutions studied. All measurements were performed at 20 °C. Interestingly, all four properties exhibited the same behavior with relaxation frequency and amplitude (illustrated in Figure 10), revealing two separate regions with distinct characteristics attributed to the structural variation taking place. These physicochemical properties were not chosen accidentally. The viscosity of a fluid is a measure of its resistance to deformation at a given rate and thus, it is a physicochemical parameter that is very sensitive to intermolecular relaxation due to the molecular associations in these solutions through hydrogen-bonding interactions. Sound velocity is an important thermo-physical property mirroring the dynamic response of the condensed phase. The velocity of sound in a liquid is sensitive to molecular changes and is dependent on the degree of order of the molecules. On the contrary, the sound absorption coefficient is sensitive to changes in particle size and molecular interactions. Mass density is very important in the investigation of concentration-induced structural changes in the liquid state. The transitions from a loose to a more rigid structure and vice versa are related to changes in the coordination and the cross-linking of interstitial spaces in the overall structure, which is reflected in the concentration dependency of the mass density. Finally, the adiabatic compressibility provides important information concerning specific interactions between the same and different molecules in the binary liquid mixtures. The expression for the adiabatic compressibility is:

(10) $$\kappa_{S}=-\frac{1}{V}{\left(\frac{\partial V}{\partial p}\right)}_{S}=\frac{1}{\rho u^{2}}$$

It is a measure of the instantaneous relative volume change of the fluid as a response to a pressure change. The results presented in Figure 11 reveal a transition from a more loosely packed structure to a more rigid one in agreement with the proposed structural model proposed based on the vibrational and acoustic spectroscopic data. The mass density and especially the kinematic viscosity data of the HMTA aqueous solutions reveal that amine molecules exert an influence on the water structural organization.

Our results are in line with the reported X-ray data that have revealed a novel type of clathrate structure, where each amine molecule occupy cavities within water clathrates constituted by hydrogen-bonded water molecules [26]. Furthermore, HMTA aqueous solutions exhibited a strong deviation from the ideal behavior suggesting the dominant role of the solute–solvent interactions [27].

## 3. Materials and Methods

### 3.1. Materials

Solid hexamethylene tetramine (HMTA) with the molecular formula (CH2)6N4 and 99% purity was obtained from Alfa Aesar and was used without further treatment or purification. At room temperature, HMTA is a white solid with ammonia-like odor. HMTA does not melt, although it sublimes at 260–295 °C. Aqueous solutions of HTMA were prepared gravimetrically in a wide concentration range using triply distilled water. All solutions were clear and colorless. Its solubility in water at 20 °C was estimated to be ~0.67 g/mL. The molal concentrations and the corresponding mole fractions are presented in Table 2. For all measurements, fresh samples were used, while special attention has been paid due to HMTA’s ability to absorb carbon dioxide.

### 3.2. Raman Spectroscopy

The procedures for acquiring polarized and depolarized Raman spectra have already reported in the past [28,29,30,31]. All samples were excited by a 532 nm continuous-wave and linearly polarized laser in a backscattering geometry. A fully integrated confocal Raman microscope instrument (Labram SoleilTM, Horiba Scientific, Palaiseau, France) equipped with 1800 gratings was used to analyze the scattered light. The analyzed light after the gratings was detected by a thermoelectrically cooled CCD camera down to −70 °C. The spectral resolution for all Raman measurements was fixed at 1.5 cm^−1^. Rayleigh scattering was reduced by means of a notch filter appropriate for the working laser wavelength. The incident power on the liquid samples was less than 3 mW to avoid thermal heating. Short accumulation times near 200 s and a cumulation number equal to 5 were sufficient to achieve Raman spectra with a high signal-to-noise ratio for all samples. Both vertical–vertical (VV) and horizontal–vertical (VH) polarization configurations were employed. A reference CCl_4_ sample was used to calibrate the monochromator’s possible drift and polarization. All Raman spectroscopic measurements were carried out at 20 °C. Quantitative Raman intensity measurements had an error that was less than 2%.

### 3.3. Ultrasonic Relaxation Spectroscopy

Ultrasonic absorption measurements were obtained in the low-frequency region under isobaric conditions by employing the transmission method [32,33,34]. A cylindrical acoustic cell was used to accommodate approximately 2 mL of the liquid sample at the desired temperature. The cell was made of fused silica glass to provide chemical durability. The temperature was controlled by an external circulation system with an accuracy of ±0.01 °C. Two identical broadband transducers (V111, central frequency 10 MHz, Olympus-Evident, Tokyo, Japan) were attached to the parallel faces of the cylindrical acoustic cell. A common medical couplant was placed between the piezoelectric element and the cell surface to ensure perfect transmission of the ultrasound. The first piezoelectric element, which acts as transmitter, was triggered by a pulse generator (TGP3151, TTi, Maisach-Gernlinden, Maisach, Germany) at the desired frequency. The time duration of the pulse was less than 3.5 microseconds, and the pulse repetition rate was fixed at 5 milliseconds. The obtained ultrasonic wave propagated back and forth through the sample many times after multiple reflections in the parallel faces of the acoustic cell. The transmitted pulse, as well as the consequential echoes, were detected by the receiving transducer and sent to a digital oscilloscope (Tektronix, TBS 1202B, Tektronix, Beaverton, OR, USA) for further signal analysis. For each frequency, the attenuation coefficient of the solution sample can be directly estimated from the exponential decrease of the signal amplitude. The accuracy of the sound absorption measurements was better than ±5%. The ultrasound speed was calculated by the time required for the ultrasonic wave to travel the fixed path length of the acoustic cell with an accuracy of ±0.01% [32,33,34]. All ultrasonic relaxation spectroscopic measurements were carried out at 20 °C.

The wave velocity was calculated based on the sample thickness inside the acoustic cell and the time separating two consecutive echoes in the back wall echo train. The amplitude of the ultrasonic wave decays exponentially with respect to the travel distance when passing through the liquid medium. The attenuation coefficient $a_{f}$ is calculated from the amplitudes of the echoes from the time domain trace as:

(11) $$a_{f}=\frac{-20}{2\left(m-n\right)d}log\left(\frac{I_{m}}{I_{n}}\right)$$

where $I_{m}$ and $I_{n}$ are the maximum amplitudes of the m-th and n-th pulse echoes, respectively.

We performed a series of sound velocity and attenuation measurements for several non-relaxing reference liquids and the results were presented in [14]. The comparison between our experimental values with the literature values revealed an adequate agreement in both velocity and sound attenuation coefficients indicating that our setup is reliable for accurately measuring the velocity and attenuation of ultrasound in liquids.

### 3.4. DFT Calculations

Quantum mechanical calculations were carried out for the HMTA and the relevant aggregated and protonated species in the gas phase using the Gaussian 09 W Revision D.01 package of programs [35]. Density functional theory (DFT) using the B3LYP functional and 6-311++G(d,p) basis set was employed to optimize the molecular structure of HMTA-based species and the corresponding vibrational frequencies [36]. Molecular volume was computed simply as the volume inside a contour of a particular electron density. Gaussian, for instance, uses the value of 0.001electrons/Bohr^3^. To determine the molecular volume, a quantum mechanical calculation is performed to obtain the wave function and then it is integrated. Finally, all the atomic volumes are summed to obtain the molecular one. More details concerning the methodology of the theoretical calculations of the reaction volume change can be found in reference [34]. We restricted our theoretical calculations in the B3LYP level of theory, which seems to be a sufficient option in terms of accuracy for the specific system, and we chose the 6-311++g(d,p) basis set due to its effectiveness when studying the hydrogen bond interactions that are present in a variety of organic compounds. The molecular structure of HMTA was downloaded as SDF digital file from the PubChem electronic database and was used for structure optimization and further calculations. The theoretically estimated vibrational spectra have been corrected with a scaling factor corresponding to the B3LYP/6-311++G(d,p) level of theory.

To elucidate the potential protonation reaction of HMTA, we performed theoretical calculations on the protonated species of HMTA with one, two, three and four protons. We chose these structures for the evaluation of the protonation reaction. To interpret the possible self-aggregation of HMTA, we performed calculations on four structures of the solute associated species (HMTA)n with aggregation number *n* = 2, 3 and 4. To clarify the hetero-aggregation mechanism of HMTA with water molecules, we constructed five aggregates HMTA·xH_2_O, x = 1–4, 20 that consist of one HMTA molecule and 1, 2, 3, 4 and 20 molecules of H_2_O, respectively. In all cases, water molecules were aggregated with the solute through hydrogen bonding between a hydrogen atom from a water molecule and a nitrogen atom from an HMTA molecule. The theoretical study of the species with the 20 water molecules surrounding the HMTA solute will provide a more realistic simulation of the dilute region. All theoretically predicted molecular structures of HMTA molecules and all HMTA protonated and aggregated species can be found in the Supplementary Material.

## 4. Conclusions

An experimental concentration-dependent Raman and ultrasonic relaxation spectroscopic study of HMTA aqueous solutions has been undertaken to study the structural changes in short- and medium-range order.

By comparing the experimental Raman spectroscopic data with quantum mechanical calculations on isolated species, the protonation of HMTA was excluded due to the extended spectral differences in the fingerprint region. In dilute solutions, the dominant mechanism is the hetero-aggregation reaction of HMTA with water, while in the intermediate concentration region, a self-aggregation mechanism is also present. Processes related with conformational changes are unlikely to occur due to the rigid molecular structure of HMTA.

The acoustic spectra of the HMTA aqueous solutions revealed a single relaxation mechanism, which was attributed to the aggregation reaction. The concentration dependence of the relaxation frequency and amplitude exhibited a drastic change below and above 0.018 mole fraction of HMTA, although with the opposite trend. This characteristic mole fraction signifies two separate regions with distinct structural characteristics. The relaxation observed in dilute solutions was attributed to the hetero-association of HMTA with water molecules, while at higher concentrations, the observed relaxation was assigned to the self-association reaction of HMTA molecules. In the low-concentration region and up to the crossover mole fraction, the relaxation amplitude decreased, while above this characteristic concentration suddenly increased indicating the strengthening of the self-aggregation of HMTA process at the expense of the hetero-aggregation mechanism of HMTA with water molecules, which is expected to be robust in the low-concentration range.

The concentration dependence of the kinematic viscosity and mass density indicate the passage from a loosely packed to a more rigid structure composed of HMTA self-aggregates. An analogous behavior was exhibited by the ultrasound velocity and the adiabatic compressibility. All four physicochemical properties experienced drastic changes when approaching the crossover mole fraction, supporting our proposed structural model proposed based on the vibrational and acoustic spectroscopic data.

## Figures and Tables

**Table 1:** Experimental and theoretical Raman frequencies of HMTA in solution and vacuum, respectively, and band assignments.

Experimental Raman Frequency (cm^−1^) in Solution	Theoretical Raman Frequency (cm^−1^) in Vacuum	Assignment	Symmetry	
459	463	CNC deformation	E	Fingerprint region (<1500 cm^−1^)
515	510	CNC deformation	F_2_	
692	680	CNC deformation	F_2_	
785	789	CNC stretching	A_1_	
823	821	CH_2_ rocking	F_2_	
1020	1019	CN stretching	F_2_	
1056	1048	CNC deformation	A_1_	
1242	1257	CN stretching	F_2_	
1353	1359	CH_2_ twisting	E	
1384	1392	CH_2_ wagging	F_2_	
1455	1483	CH_2_ deformation	E	
2902	3037	Symmetric and asymmetric C-H stretching		C-H region
2931	3039			
2959	3052			
2988	3088			
3253	-	Stretching of O-H of water molecules		O-H region
3410	-	Stretching of O-H of water molecules		
3636	-	Stretching of O-H of water molecules that are hydrogen bonded with HMTA molecule		
3648	-	Stretching of O-H of water molecules		

**Table 2:** Molal concentrations and the corresponding mole fractions.

Molal Concentration (mol/Kg_solvent_)	Mole Fraction
1 × 10^−5^	1.80 × 10^−7^
1 × 10^−4^	1.80 × 10^−6^
1 × 10^−3^	1.80 × 10^−5^
0.01	1.80 × 10^−4^
0.1	1.82 × 10^−3^
1.0	0.018
2.0	0.035
3.0	0.051
4.0	0.067
5.0	0.083

## Data Availability

Data are available upon request to the corresponding author.

## Supplementary Materials

The following supporting information can be downloaded at: https://www.mdpi.com/article/10.3390/molecules28237838/s1, Figures S1–S9: Theoretically predicted molecular structures of HMTA molecule and all HMTA protonated and aggregated species.

## References

[1] Bakhit M., Krzyzaniak N., Hilder J., Clark J., Scott A.M., Mar C.D. (2021). Use of methenamine hippurate to prevent urinary tract infections in community adult women: A systematic review and meta-analysis. Br. J. Gen. Pract..

[2] Schoen A.H. (2004). Re: Equivalence of methenamine Tablets Standard for Flammability of Carpets and Rugs. Report of U.S. Consumer Product Safety Commission. https://www.federalregister.gov/documents/2007/10/26/E7-20666/technical-amendment-to-the-flammability-standards-for-carpets-and-rugs.

[3] Retrieved from UK Food Standards Agency: “Current EU Approved Additives and Their E Numbers”. https://www.food.gov.uk/business-guidance/approved-additives-and-e-numbers.

[4] Eller K., Henkes E., Rossbacher R., Höke H. (2000). Amines, Aliphatic. Ullmann’s Encyclopedia of Industrial Chemistry.

[5] Craik D.J., Levy G.C., Lombardo A. (1982). Carbon-13 and nitrogen-15 nuclear magnetic resonance of polycyclic polyamines. A study of solution nitrogen-hydrogen hydrogen bonding and protonation. J. Phys. Chem..

[6] Tryfon A., Siafarika P., Kouderis C., Kaziannis S., Boghosian S., Kalampounias A.G. (2023). Evidence of Self-Association and Conformational Change in Nisin Antimicrobial Polypeptide Solutions: A Combined Raman and Ultrasonic Relaxation Spectroscopic and Theoretical Study. Antibiotics.

[7] Kalampounias A.G., Yannopoulos S.N., Steffen W., Kirillova L.I., Kirillov S.A. (2003). Short-time dynamics of glass-forming liquids: Phenyl salicylate (salol) in bulk liquid, dilute solution, and confining geometries. J. Chem. Phys..

[8] Tsigoias S., Kouderis C., Mylona-Kosmas A., Boghosian S., Kalampounias A.G. (2020). Proton-transfer in 1,1,3,3 tetramethyl guanidine by means of ultrasonic relaxation and Raman spectroscopies and molecular orbital calculations. Spectrochim. Acta A.

[9] Tsigoias S., Papanikolaou M.G., Kabanos T.A., Kalampounias A.G. (2021). Structure and dynamics of aqueous norspermidine solutions: An in situ ultrasonic relaxation spectroscopic study. J. Phys. Condens. Matter.

[10] Bernasconi C.F. (1976). Relaxation Kinetics.

[11] Risva M., Siafarika P., Kalampounias A.G. (2022). On the conformational equilibria between isobutyl halide (CH_3_)_2_CH–CH_2_X (X=Cl, Br and I) rotational isomers: A combined ultrasonic relaxation spectroscopic and computational study. Physica B.

[12] Mpourazanis P., Stogiannidis G., Tsigoias S., Kalampounias A.G. (2019). Transverse phonons and intermediate-range order in Sr-Mg fluorophosphate glasses Spectrochim. Acta A.

[13] Mpourazanis P., Stogiannidis G., Tsigoias S., Papatheodorou G.N., Kalampounias A.G. (2019). Ionic to covalent glass network transition: Effects on elastic and vibrational properties according to ultrasonic echography and Raman spectroscopy. J. Phys. Chem. Solids..

[14] Stogiannidis G., Tsigoias S., Mpourazanis P., Boghosian S., Kaziannis S., Kalampounias A.G. (2019). Dynamics and vibrational coupling of methyl acetate dissolved in ethanol. Chem. Phys..

[15] Ivanov E.V., Batov D.V. (2019). Unusual behavior of temperature-dependent solvent H/D isotope effects in the enthalpy and heat capacity of hexamethylenetetramine (urotropine) hydration. J. Molec. Liq..

[16] Ivanov E.V. (2018). Temperature-dependent standard volumetric properties of hexamethylenetetramine in ordinary and deuterated water: A study resolving debatable issues being commented in the [Journal of Molecular Liquids, 248 (2017) 48–52]. J. Molec. Liq..

[17] Thomas C.W. (1965). Hexamethylenetetramine Hexahydrate: A New Type of Clathrate Hydrate. J. Chem. Phys..

[18] Risva M., Tsigoias S., Boghosian S., Kaziannis S., Kalampounias A.G. (2023). Exploring the influence of urea on the proton-transfer reaction in aqueous amine solutions with Raman and ultrasonic relaxation spectroscopy. Mol. Phys..

[19] Chettiyankandy P., Chand A., Ghosh R., Sarkar S.K., Das P., Chowdhuri S. (2019). Effects of hexamethylenetetramine (HMTA) on the aqueous solution structure, dynamics and ion solvation scenario: A concentration and temperature dependent study with potential HMTA models. J. Molec. Liq..

[20] Santos P.S. (1989). Raman Spectra of Some Adducts of Hexamethylenetetramine. J. Raman Spectr..

[21] Bertie J.E., Solinas M. (1974). Infrared and Raman spectra and the vibrational assignment of hexamethylenetetramine-h12 and -d12. J. Chem. Phys..

[22] Litovitz T.A., Davis C.M., Mason W.P. (1965). Physical Acoustics.

[23] Herzfeld K.F., Litovitz T.A. (1959). Absorption and Dispersion of Ultrasonic Waves.

[24] Ensminger D., Bond L.J. (2011). Ultrasonics: Fundamentals, Technologies, and Applications.

[25] Blandamer M.J. (1973). Introduction to Chemical Ultrasonics.

[26] Davidson D.W. (1968). Dielectric relaxation and orientational ordering of water molecules in hexamethylenetetramine hexahydrate. Can. J. Chem..

[27] Crescenzi V., Quadrifoglio F., Vitagliano V. (1967). Hexamethylenetetramine Aqueous Solutions. Isopiestic Data at 25° and Density and Viscosity Data in the Range 3-34o. J. Phys. Chem..

[28] Kalampounias A.G., Tsilomelekis G., Boghosian S. (2015). Glass-forming ability of TeO_2_ and temperature induced changes on the structure of the glassy, supercooled, and molten states. J. Chem. Phys..

[29] Kalampounias A.G., Yannopoulos S.N., Papatheodorou G.N. (2006). Temperature- induced structural changes in glassy, supercooled, and molten silica from 77 to 2150 K. J. Chem. Phys..

[30] Latsis G.K., Banti C.N., Kourkoumelis N., Papatriantafyllopoulou C., Panagiotou N., Tasiopoulos A., Douvalis A., Kalampounias A.G., Bakas T., Hadjikakou S.K. (2018). Poly Organotin Acetates against DNA with Possible Implementation on Human Breast Cancer. Int. J. Mol. Sci..

[31] Kalampounias A.G., Kirillov S.A., Steffen W., Yannopoulos S.N. (2003). Raman spectra and microscopic dynamics of bulk and confined salol. J. Mol. Struct..

[32] Kouderis C., Siafarika P., Kalampounias A.G. (2021). Disentangling proton-transfer and segmental motion relaxations in poly-vinyl-alcohol aqueous solutions by means of ultrasonic relaxation spectroscopy. Polymer.

[33] Kalampounias A.G. (2022). Establishing the role of shear viscosity on the rate constants of conformational fluctuations in unsaturated aldehydes. Chem. Phys..

[34] Kouderis C., Siafarika P., Kalampounias A.G. (2021). Molecular relaxation dynamics and self-association of dexamethasone sodium phosphate solutions. Chem. Pap..

[35] Frisch M.J., Trucks G.W., Schlegel H.B., Scuseria G.E., Robb M.A., Cheeseman J.R., Scalmani G., Barone V., Petersson G.A., Nakatsuji H. (2009). Gaussian 09, Revision A.02.

[36] Becke A. (1993). Density-Functional Thermochemistry. III. The Role of Exact Exchange. J. Chem. Phys..

