# Supplementary material for: Identification of Aggregation Processes in Hexamethylenetetramine Aqueous Solutions: A Comprehensive Raman and Acoustic Spectroscopic Study Combined with Density Functional Theory Calculations
Source: Molecules. 2023 Nov 29;28(23):7838. doi: 10.3390/molecules28237838 (PMC10707806; doi:10.3390/molecules28237838)
Supplement: Supplementary file 1 [file molecules-28-07838-s001.zip › molecules-2720502-supplementary.pdf]

Supplementary Material

Theoretically predicted molecular structures of HMTA molecule and all HMTA protonated and aggregated species. All quantum mechanical calculations were performed by means of B3LYP/6-311++g(d,p) method. Interatomic distances are also shown. See main text for more details concerning the theoretical calculations.

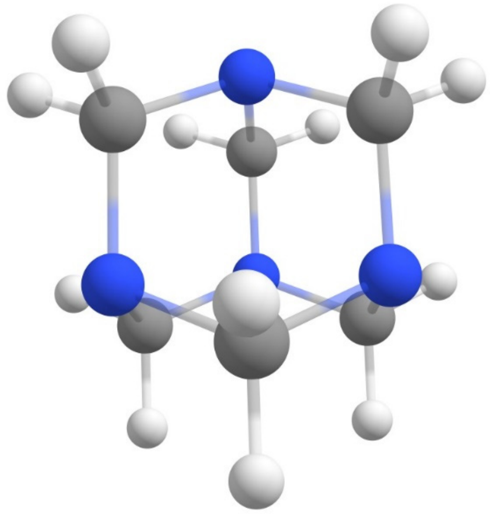

Figure S1. The optimized molecular structure of HMTA molecule.

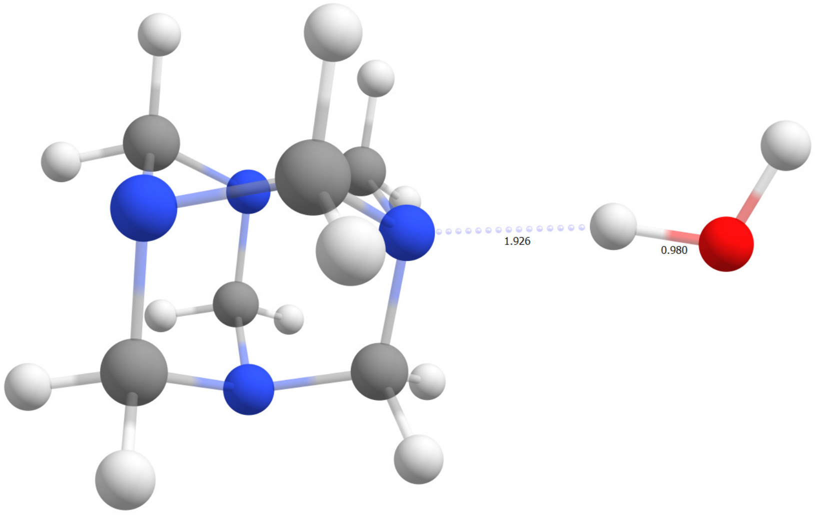

Figure S2. The optimized molecular structure of the HMTA·H_2O aggregate with one water molecule.

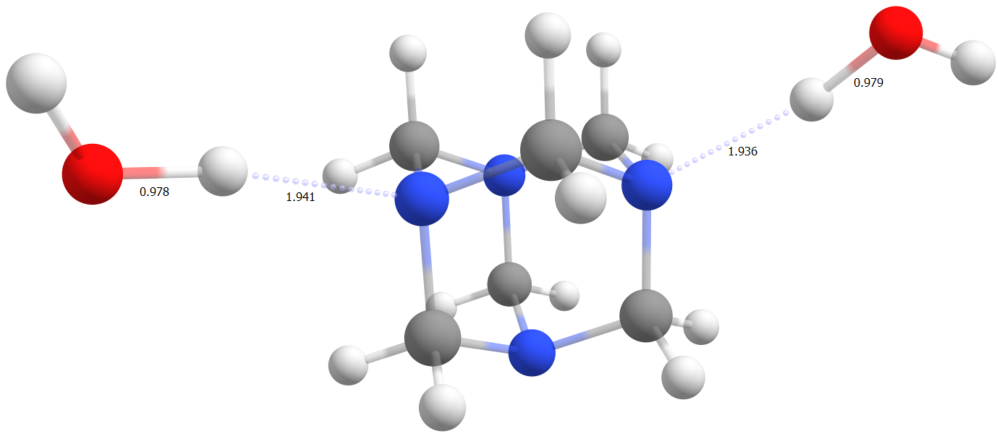

Figure S3. The optimized molecular structure of the HMTA·$2H_2O$ aggregate with two water molecules.

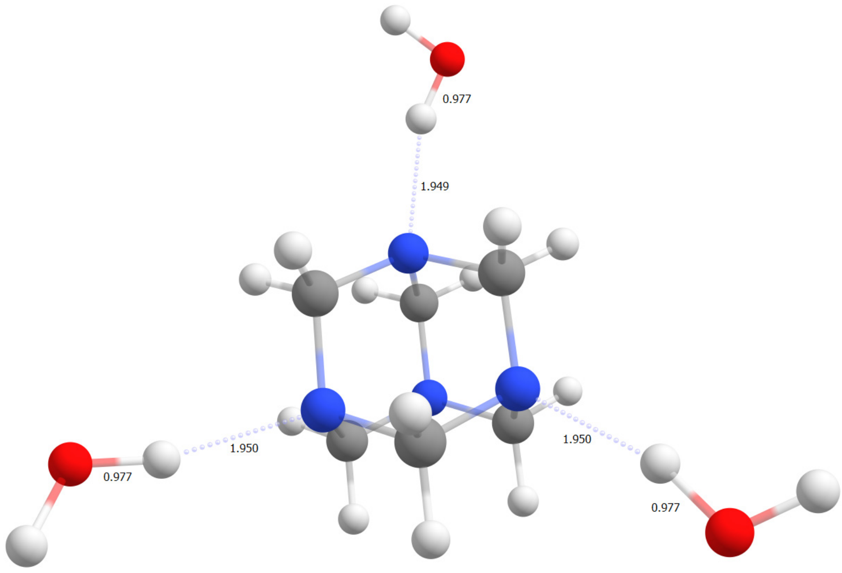

Figure S4. The optimized molecular structure of the HMTA·$3H_2O$ aggregate with three water molecules.

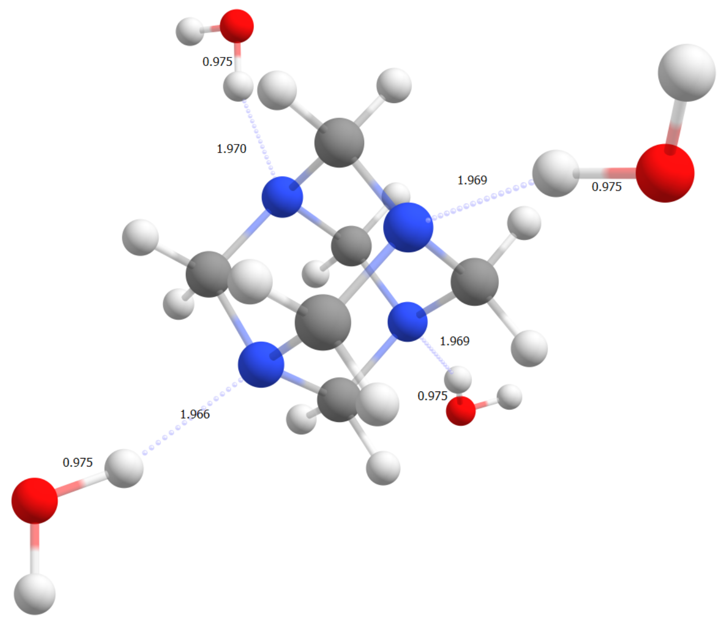

Figure S5. The optimized molecular structure of the HMTA·$4H_2O$ aggregate with four water molecules.

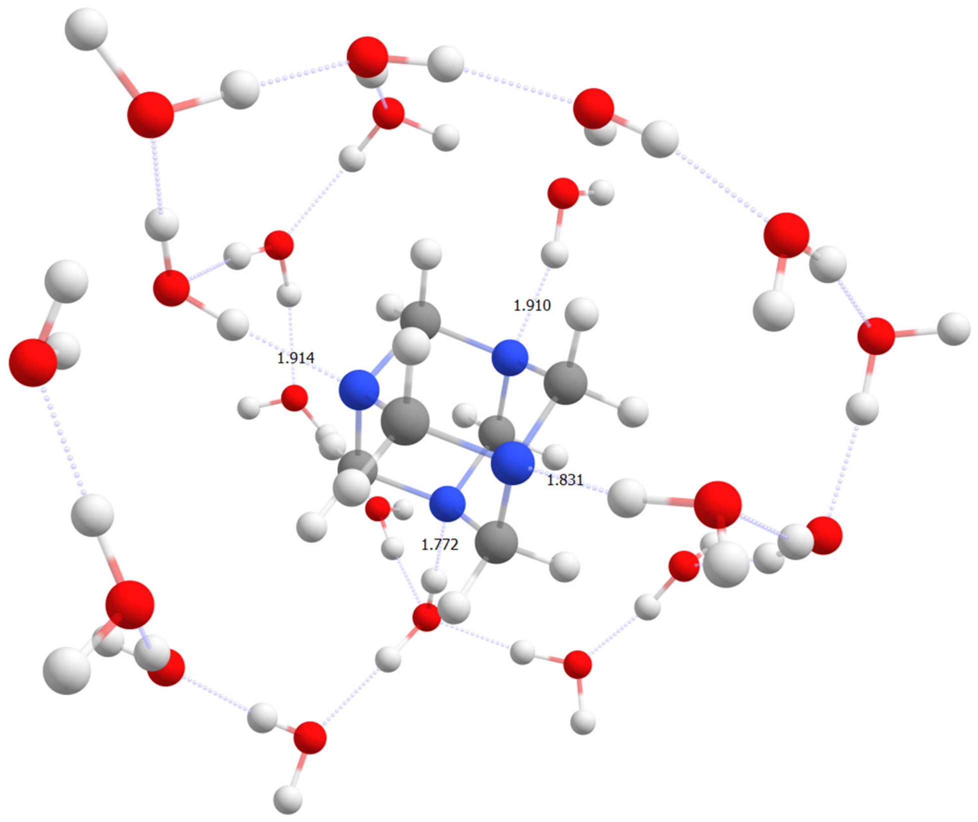

Figure S6. The optimized molecular structure of the HMTA·$20H_2O$ aggregate with twenty water molecules.

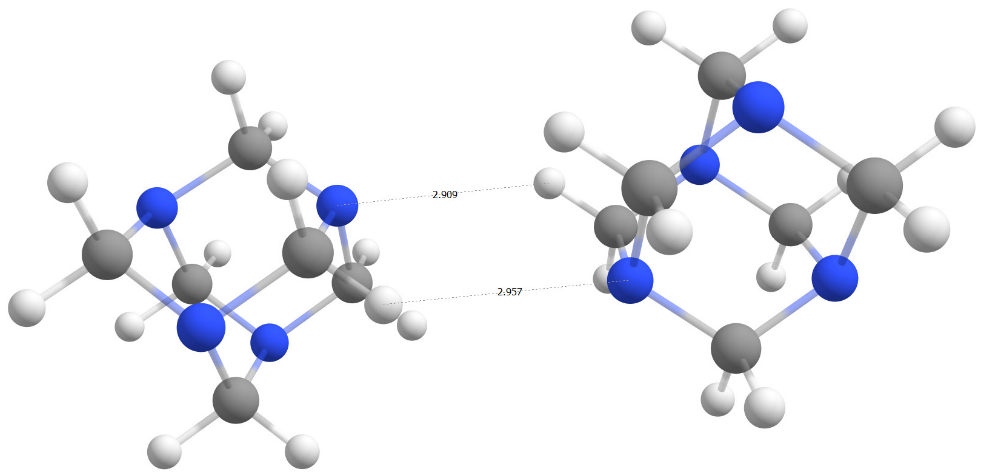

Figure S7. The optimized molecular structure of the $(HMTA)_2$ dimer.

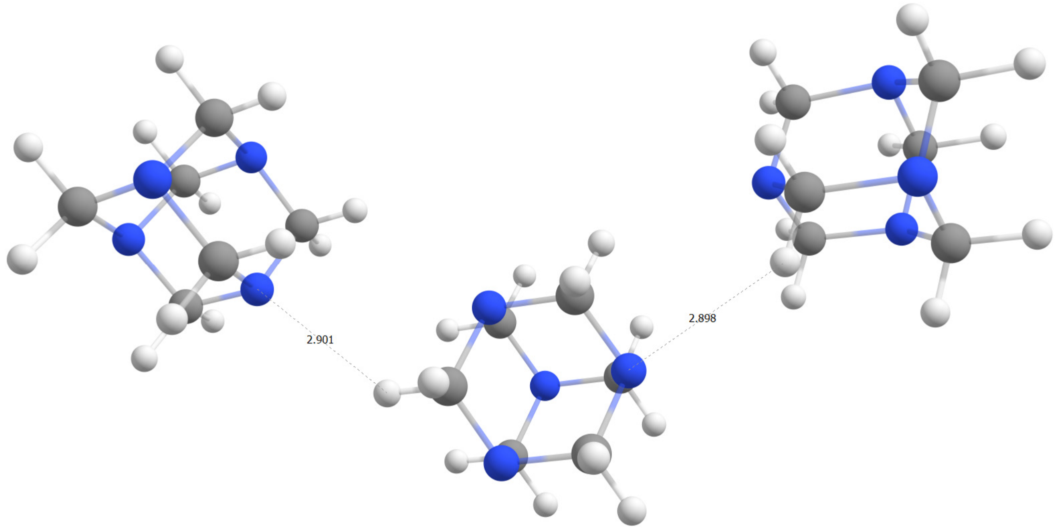

Figure S8. The optimized molecular structure of the $(HMTA)_3$ trimer.

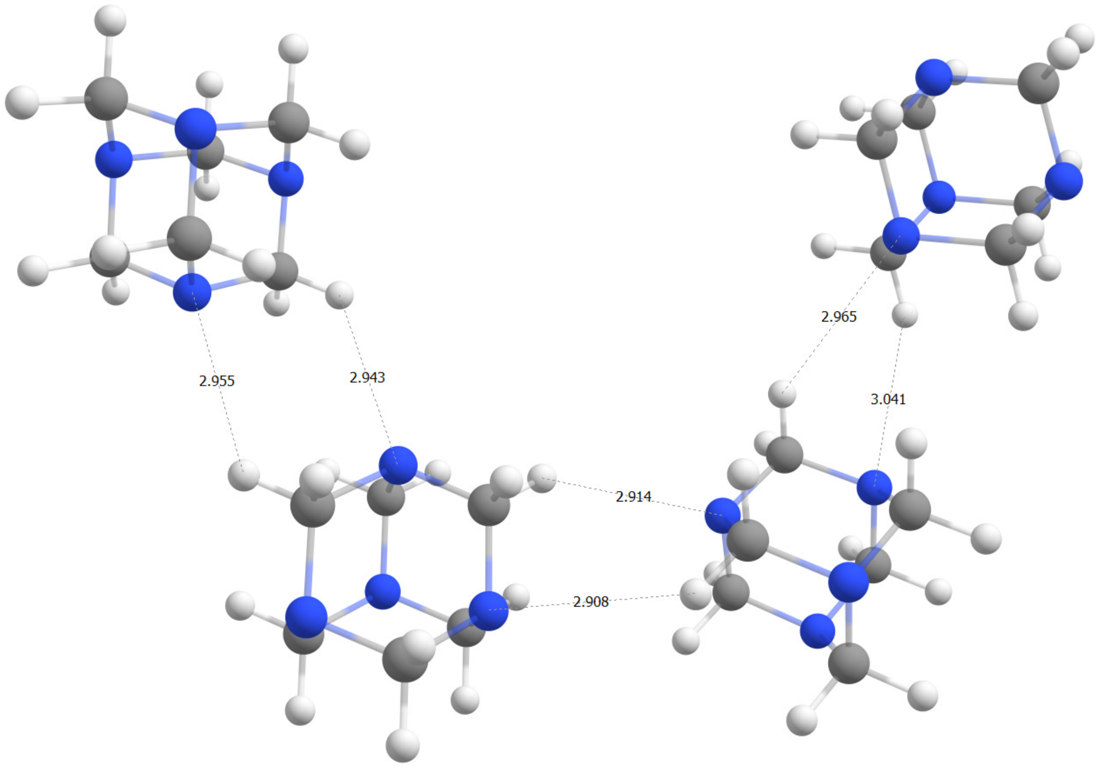

Figure S9. The optimized molecular structure of the $(HMTA)_4$ tetramer.
